# Supplementary material for: Are carbon emissions trading and green financial instruments synergistic? -Comprehensive quantitative research based on content analysis
Source: PLoS One. 2024 Mar 7;19(3):e0298601. doi: 10.1371/journal.pone.0298601 (PMC10919601; doi:10.1371/journal.pone.0298601)
Supplement: S1 Data — (ZIP) [file pone.0298601.s001.zip › Data/地级市Z_F工作报告.zip!/╕╩╦α/╒┼╥┤╩╨/└┤╫╘╣1⁄2╓┌║┼í╛┬φ┐╦╩2╛▌═°í┐_2017.docx]

各位代表：

现在，我代表市人民政府向大会报告工作，请予审议，并请各位政协委员和列席人员提出意见。

本届政府工作回顾

五年来，我们在省委、省政府和市委的坚强领导下，认真贯彻落实党的十八大及十八届三中、四中、五中、六中全会和习近平总书记系列重要讲话精神，按照市委“坚定贯彻‘五大发展理念’，围绕‘四二’部署，实施‘六六’战略，加快建设幸福美好金张掖”的基本思路，牢牢把握稳中求进总基调，积极适应经济发展新常态，统筹推进稳增长、促改革、调结构、惠民生、防风险各项工作，较好地完成了“十二五”规划及市三届人大一次会议确定的各项目标任务，经济社会呈现出持续稳定健康发展的良好局面。

——综合实力迈上新台阶。主要经济指标增速高于全省、全国平均水平，在全省的位次实现前移。预计2016年实现生产总值396亿元，完成固定资产投资350亿元，招商引资到位资金500亿元，比2011年分别增长54.2%、141%和600%。大口径财政收入、一般公共预算收入达到58.3亿元和26.6亿元，比2011年增长133%和158%。存款余额突破600亿元，比2011年增长185%；存贷比达到96%，提高44.6个百分点。国家玉米制种基地、国家现代农业示范区、全国主体功能区建设试点示范市等9个国家级战略平台落地建设。经济实力、基础条件、发展活力明显增强，区域发展基础更加坚实。

——产业培育取得新突破。积极挖掘特色优势资源开发潜力，主导产业持续壮大。农业固定资产投资比2011年增长2.72倍，农业增加值位居全省第一；“2+工业园区体系基本形成，规上工业企业数实现翻番；旅游文化产业异军突起，旅游接待人数和综合收入比2011年增长4.7倍和4.8倍。三次产业比重由2011年的26.5∶34.8∶38.7调整为25.3∶27.8∶46.9，三产占比提高8.2个百分点。经济结构更趋合理，转型发展的内生动力和协调性、稳定性、可持续性进一步增强。

——发展动能实现新转换。创新驱动战略深入实施，科技创新对经济的支撑引领作用明显增强。全国首批小微企业创业创新基地示范城市加快建设，创业创新成为鲜明导向。万人口发明专利拥有量、科技研发投入占GDP比重、科技成果转化率全面上升，科技对经济增长的贡献率达到51%，比2011年提高5个百分点。单位GDP能耗、水耗、主要污染物排放量全面下降。农村产权制度改革、投融资体制改革、行政审批制度改革、商事制度改革等重大改革稳步推进，改革创新成为经济社会发展新动能。

——城乡建设呈现新面貌。城市建成区面积达到95.9平方公里，比2011年翻了近一番，两个“1+5”城市框架体系基本形成。城镇化率达到43.7%，比2011年提高7.7个百分点。市区和各县城集中供热供水、垃圾收集、污水处理设施建成投用，城市聚集要素、辐射农村的功能不断增强。以“美丽乡村”和“四化”新农村为引领，建成一批设施较为完善、地域特色鲜明的示范村。公路、电力、通讯实现村村通，网络实现乡镇全覆盖，在全省率先建成“全光网”市，城乡面貌发生深刻变化。

——人民生活达到新水平。城镇、农村居民人均可支配收入持续稳定增长，预计今年达到21250元和11800元，年均增长10.7%和12.3%。精准扶贫扎实推进，累计投入扶贫资金20亿元，12.5万人实现稳定脱贫，贫困发生率由2011年的13.21%下降到2%以内。民生支出占公共预算的比例提高到85%，五年累计投入541亿元。新建成各级各类学校、医院、幼儿园、养老中心443所，总投资达20.56亿元。城乡居民教育、医疗、养老、文化服务、社会保障水平显著提高。

五年来，我们主要做了以下工作：

（一）坚持抓项目、扩投资，发展动力日趋强劲。紧紧抓住项目建设这个“牛鼻子”，围绕国家产业政策、区域经济政策和投资导向，谋划、论证、争取、实施了一批带动能力强、事关可持续发展的大项目、好项目。五年来，累计实施各类项目2880多项，完成固定资产投资1341亿元，比前五年增长1.6倍。争取中央预算内投资41.23亿元、省级投资3.89亿元。张掖机场、兰新高铁、市区热电联产集中供热、黑河湿地湖泊治理、湿地生态治理与恢复、国家湿地公园、丹霞七彩镇、丹霞通用航空机场、市级“三馆”等一批事关全局和长远发展的重大项目顺利建成，兰州至张掖客运专线、张扁高速、750千伏张掖输变电等重点项目前期工作有序推进。大力实施“引强入张”战略，招商引资到位资金连续四年实现百亿元级增长，总额达到1628亿元。红星美凯龙、碧桂园、黄河矿业、长城影视等知名企业落户张掖，与中铁建、甘建投等大型集团合作取得实质性进展。民间投资持续活跃，占比达到65%，对税收、就业的贡献率不断提高。组建市级PPP项目中心，建立市级PPP项目库，入库项目624项，纳入国家、省级项目库68项，政府与社会资本合作的渠道进一步拓展。

（二）坚持抓质量、提效益，产业特色更加鲜明。突出协调发展、转型升级，加快培育壮大主导产业，质量和效益进一步提升。

现代农业优势彰显。坚持“绿色、循环、效益、品牌”发展理念，持续培育壮大优势主导产业和区域特色产业规模，扎实推进玉米制种、奶肉牛养殖、绿色有机蔬菜、优质饲草“四个百万”工程，建成一批规模大、标准化程度高的生产基地，形成100万亩玉米制种、100万头奶肉牛、61万亩高原夏菜、45万亩马铃薯、42万亩中药材、40万亩优质林果、136万亩饲草、3万吨食用菌产业规模。全省现代畜牧业全产业链试点工作扎实推进，全国粮改饲和草牧业试点等一批重点项目获批实施，六县区全部进入全省牛羊产业大县行列。农产品加工产业链基本形成，沅博、博峰、前进牧业、金阳光等96户龙头企业建成投产，成都菊乐、三元乳业等大型养殖和高端乳制品加工企业落地建设。建成善之荣食用菌产业园、德隆10万吨果蔬恒温库等投资千万元以上的农业产业化项目80个。新型经营主体增长18倍，达到2.3万户。农产品加工转化率提高6个百分点，达到61%。种植业、养殖业、菌业“三元双向”循环模式基本形成，农业比较优势进一步巩固提升。全力打造“金张掖北纬38º”地域品牌，“张掖玉米种子”“张掖肉牛”等10个产品获全国地理标志证明商标、农产品地理标志登记和地理标志产品保护。甘州、临泽命名为全省农产品质量安全县区。农业“走出去”战略加快推进，“金花寨”小米和一批绿色有机蔬菜出口中亚和非洲地区。

生态工业稳步发展。坚持资源循环、企业聚集的生态工业发展之路，加强要素保障，优化服务环境，壮大产业集群，工业经济保持稳定增长的良好态势。张掖工业园区晋升为国家级经济技术开发区，民乐生态工业园区和4个省级园区基础设施日臻完善，国电龙源、上海航天、青龙管业等967户各类企业落户园区，聚集带动效应持续提升。把绿色有机食品加工业作为工业经济发展的主攻方向，以特色农产品精深加工为支撑、新能源新材料等战略性新兴产业为牵引的“一特两新”工业经济体系初步建立，形成300万吨农产品加工、200万吨有色金属矿选生产能力和200万千瓦发电装机容量。新增规上企业129户，达到223户，工业增加值年均增长10%。培育全国驰名商标4件、甘肃名牌产品32个，滨河集团获省政府质量奖。整合关闭煤矿68户，淘汰落后产能230万吨。

旅游文化产业迅猛发展。着力打造丝绸之路黄金旅游线重要目的地、中国西部区域游客集散中心和国际特色休闲度假名城，做靓做响地貌景观大观园、暑天休闲度假城、丝绸之路古城邦、户外运动体验区、西路军魂传承地“五张名片”，深入开展“四大行动”，旅游业“井喷式”发展，接待游客突破2000万人次，实现旅游综合收入100亿元，直接就业4万人。旅游景区晋等升级成效明显，新增国家级景区10家，总数达到15家，丹霞景区跻身全国百万人次大景区行列。丹霞国家地质公园、平山湖大峡谷、大湖湾文化旅游风景区、中华裕固风情走廊、河西民俗文化村、方舟水世界等80多个重点项目建成运营。旅游文化全产业链打造初具形态，张掖被确定为全国体育产业联系点和全省旅游文化体育医养融合发展示范区，国际户外运动名城建设列入国家体育总局规划；中国·张掖汽车拉力锦标赛、祁连山国际超百公里山地户外运动挑战赛等品牌赛事对外影响力持续扩大；祁连玉文化产业园命名为国家级文化产业示范基地，文化产业增加值占GDP比重提高1.5个百分点，增速居全省前列。旅游管理服务水平明显提升，组建丹霞大景区管委会，成立旅游警察支队，综合执法力量得到加强，管理服务人员培训实现常态化，市场秩序集中整治力度持续加大，监管服务水平显著提高。旅游全民宣传广泛开展，“湿地之夏·金张掖”旅游文化艺术节、绿洲论坛、“张交会”、首届丝绸之路国际通用航空大会等重大节会成功举办；成立河西五市及最美国道227线旅游联盟，开通上海、杭州等地旅游包机和“金张掖”号、“张掖丹霞”号等品牌列车；百万元全民宣传奖颁发两届，张掖旅游的影响力和美誉度持续提升。

商贸流通体系日趋完善。建成国家级玉米种子产业园和电子交易平台，绿洲4S汽贸城、嘉信农产品市场等128个重点商流项目建成投用。发展电商企业和网店商家1200多户，交易额达57亿元。淘宝·特色中国张掖馆上线运营，临泽、山丹列为国家电子商务进农村综合示范县。金融保险、健康养老、快递服务等新兴业态快速发展。第三产业增加值、税收、固定资产投资占比稳步提高，成为推动转型发展最活跃、最有力的因素。

（三）坚持抓改革、促开放，创新驱动更具活力。全面深化改革开放，重点领域和关键环节改革取得新突破。

行政审批制度改革稳步推进。先后9次取消、调整、下放行政审批事项483项，216项非行政许可审批事项全部取消。“营改增”政策全面落实。环评审批制度改革试点取得重大突破，评估类项目大幅减少，审批时间大幅缩短，企业负担明显减轻，监管工作全面加强。

重点领域改革持续深化。农村土地承包经营权确权登记颁证全面推进，集体土地所有权、宅基地和集体建设用地使用权登记发证工作基本完成，集体林权制度改革及配套改革成效显现。建成全省首家农村产权交易中心，发放产权融资抵押贷款9.13亿元。临泽被确定为全国不动产统一登记信息平台建设联系点和全省不动产统一登记试点县。流转土地122万亩，占二轮土地承包面积的49%，高于全省平均水平26个百分点。“五证合一”“一照”等商事制度改革措施全面落实，新发展市场主体6.8万户，新增注册资金1042亿元。全面推进公共资源配置市场化改革，市县公共资源交易中心建成运行，累计完成各类交易9945项386.5亿元。市县两级国企改革全面启动。工商质监、食品药品、卫生计生、文化广电等管理体制进一步理顺，市级检验检测认证机构整合工作基本完成。机关事业单位养老保险、县级以下公务员职务与职级并行、市级公车改革全面完成。

投融资体制改革取得重大突破。新组建山水文旅投、交投、农投、水投4家政府投融资平台，与城投集团形成相互支撑、互为借力的发展格局，资产总额达到135亿元，实现融资63亿元。与农发行、国开行签订战略合作协议，授信额度1100亿元。争取国家专项基金10.85亿元、政府债券资金35.1亿元。11亿“14张掖债”成功发行。在全省率先出台培育多层次资本市场支持企业扩大直接融资办法，山水文旅投成为甘肃旅游产业投资基金主发起单位，首期募集资金6亿元。华瑞农业在新三板挂牌，325户企业在甘肃、上海等股权交易市场挂牌。甘肃银行、兰州银行和华安证券在张设立分支机构。

“双创示范”城市建设取得阶段性成果。成功入围全国首批15家“双创示范”城市，获得国家支持资金6亿元。建成各类创业创新基地35个，总面积达84万平方米，入驻入孵企业938户；引进高层次人才235名，聘请创业导师200多人，成立西部创客大学，两个“1+3”服务体系加快构建；制定出台“19+1”优惠政策，发放各类奖补资金1.6亿元，新增小微企业11391户，直接拉动小微企业和创业者投资21亿元。实施各类科技项目348项，科技研发投入占GDP比重达到1.18%，培育高新技术企业16家，专利申请量、授权量达到4592件、1468件，是2011年的7.3倍和3.8倍，科技创新驱动力明显增强。

（四）坚持抓基础、固根本，发展后劲持续增强。大力推进市政、交通、水利、电力、信息、生态等基础设施建设，发展基础更加稳固。

城乡基础设施不断完善。累计实施各类城建项目488项，完成投资116.8亿元。城区供水供热、污水处理、垃圾收集实现全覆盖。滨河新区路网、管网及水电气暖保障设施基本建成，张掖宾馆、大成学校、甘州妇幼保健院等一批公共服务项目建成投用，综合承载功能日趋完善。稳步推进城乡一体化，高台国家新型城镇化综合试点和甘州党寨、山丹位奇省级试点工作进展有序，甘州沙井、高台南华等12个国家级重点镇加快建设，建成“美丽乡村”117个。

交通基础设施建设全面提速。新改建等级公路，累计完成投资60亿元。建制村通畅率达100%，公路通车总里程达到1.。张肃二级公路、丹霞景区快速通道、六东一级公路一期等一批重大交通项目相继建成。

水利基础设施建设力度持续加大。红山湾水库等重点水源工程建成投用，河西走廊国家级高效节水灌溉、黑河张掖城区段治理、大中型灌区节水改造、农村饮水安全等重点水利项目加快建设，完成投资47亿元。整理高标准农田79万亩。

电力和通讯基础设施建设加快推进。新建330、110千伏变电站10座，改建输变电线路。张掖被列为国家智慧城市试点市，成功入选“宽带中国”示范市和农村宽带普遍服务补偿机制试点市，信息基础设施建设达到全国领先水平。

生态保护建设走在全国前列。实施各类生态项目522项，完成投资321.3亿元。国家生态文明建设示范市、全国水生态文明建设试点市加快建设，黑河湿地国家级自然保护区总体规划、祁连山生态保护与建设综合治理规划获批，黑河流域综合治理规划通过评审，编制完成丝绸之路经济带黑河流域湿地生态走廊建设规划。黑河湿地列入国际重要湿地名录。新增森林面积49.34万亩，森林覆盖率达到17.3%。创建国家园林县城2个，荣获全国绿化模范城市和全省卫生城市称号。积极推进城乡环境整治，拆除城区燃煤小锅炉207台，大气污染治理成效明显，生产生活生态环境不断改善。

（五）坚持抓保障、惠民生，社会事业全面进步。落实创业就业各项优惠政策，五年城镇新增就业17.55万人。五大社会保险参保人数稳步增加，城镇居民基本医疗保险和城乡居民基本养老保险参保率达到99%和97.6%。新农合报销比例由2011年的50.33%提高到63.39%，城乡低保标准提高181%和260%，五保对象分散和集中供养标准提高124%和132%。建成保障性安居工程54987套，改造农村危旧房12.7万户，完成312.既有建筑节能改造。新建、改扩建幼儿园215所、义务教育学校206所，五县区通过义务教育发展基本均衡县国家评估认定，高考升学率稳居全省前列。张掖医专、市医院并入河西学院，部分医学专业实现“专升本”，培黎国际职业学院开工建设。市第二人民医院、甘州全国综合养老示范基地、市老年人康复中心等重点项目加快建设。县级公立医院综合改革有序推进，重大疾病防控工作成效明显，医疗保障水平不断提高。计划生育优惠政策全面落实，人口工作水平持续提升。深入推进华夏文明传承创新区建设，成功创建国家公共文化服务体系示范区，完成公共文化服务投资24.8亿元，湿地博物馆、丹霞地质博物馆等63个重点文化场馆及60个乡镇综合文化站、835个村文化活动室、735个“乡村舞台”建成投用。大佛寺、山西会馆修缮和汉明长城防洪等39项重点文物保护工程全面完成，历史文化名城得到保护提升。一批本土特色文艺作品多次赴国境外演出并获奖。建成各类体育设施855处，全民健身活动广泛开展。扎实推进精准扶贫脱贫，5个“插花型”贫困县区全部摘帽，65个贫困村实现整村脱贫。双拥共建、外事侨务、机构编制、审计统计 、物价粮食、老龄妇幼、人防气象、史志档案等工作都取得了新的明显进步。

（六）坚持抓作风、转机制，政府效能不断提升。坚持依法行政，全面落实省委、省政府和市委各项决策部署，严格执行政府决策议事规则和工作程序。主动接受人大法律监督和工作监督，自觉接受政协民主监督、职能机关专门监督和社会各界舆论监督，五年办结人大代表意见建议509件、政协委员提案754件，办结率100%。进一步完善政府法律顾问制度，重大事项社会听证、公示等制度得到较好落实。认真落实政府系统全面从严治党主体责任，严格贯彻执行中央八项规定精神、省委“双十条”规定和市委八项要求，扎实开展党的群众路线教育实践活动、“三严三实”专题教育和“两学一做”学习教育，政府自身建设持续加强。全面公开财政预决算、部门预算、“三公”经费，财政资金管理使用规范高效。调整腾退办公用房6万多平方米。深入推进政务公开和电子政务，实行行政审批事项网上“一站式”办结和联审联批，“三张清单一张网”建立并上线发布，权力运行更加规范透明。社会稳定风险评估和农村社区化、社区网格化管理等工作走在全省前列，5县区被命名为全省“平安县区”。建立严格的市县乡三级食品药品监管体系，甘州、高台被列为全省首批食品安全创建试点县。加大民族地区支持力度，依法管理民族宗教事务。扎实推进法治社会建设，被评为全国“六五”普法先进市。健全完善矛盾纠纷多元排查调处、依法信访工作机制，扎实开展安全隐患排查整治专项行动，信访秩序、安全生产形势持续好转。完善应急管理体系，防灾减灾救灾能力明显提高。公民文明素质不断提升，“平安张掖”建设成效明显，社会大局和谐稳定。

过去五年，张掖的经济社会发展取得了新的进步和成就，转型升级步伐加快，城乡面貌深刻变化，对外影响力持续扩大，人民生活水平不断提高。这些成绩的取得，是市委坚强领导、人大政协全力支持的结果，是全市各族人民开拓创新、苦干实干的结果。在此，我代表市人民政府，向关心、支持张掖发展的社会各界人士，向奋战在各条战线上的人大代表、政协委员、干部群众，向各民主党派、工商联、人民团体、中央和省属驻张单位，向离退休老同志、驻张部队、武警官兵、公安干警表示衷心的感谢并致以崇高的敬意！

五年的艰苦努力和奋斗历程，使我们深深体会到，要把张掖的事情办好，必须始终坚持党的领导，自觉维护中央和省、市委权威，高效贯彻落实各项决策部署；必须始终坚持发展第一要务，做大经济总量，转变发展方式，提高质量效益；必须始终坚持改革创新，着力破解体制机制障碍，以改革激发活力，以创新引领发展；必须始终坚持依法行政，强化法治理念，规范行政行为，提升政府公信力；必须始终坚持勤政为民，多谋民生之利、多解民生之忧、多办民生之事，让人民群众得到更多实惠。这些经验弥足珍贵，我们既要始终坚持、发扬光大，更要与时俱进、不断创新。

各位代表，在总结成绩的同时，我们也清醒地认识到，经济社会发展中还存在诸多困难和不足。经济下行压力大，科技创新能力不足，结构调整、转型升级、提质增效的任务还很艰巨；市场和环境双重约束趋紧，工业项目、投资、产值增长缓慢；财源培植乏力，非税收入占比过高，财政收支矛盾仍很突出；城乡基础设施仍不完善，建设管理水平与群众的期待还有一定差距；农业集约化水平不高、产业链不健全，农业持续增效、农民稳定增收难度加大，城镇居民收入仍低于全国、全省平均水平，消费拉动和内生发展动力仍然较弱；政府职能转变还不到位，一些干部担当精神、责任意识不强，行政效率和服务质量还不能适应形势发展需要。这些问题，需要在今后工作中采取针对性措施，切实加以解决。

未来五年的目标任务

新的五年，是率先在全省实现全面小康、建设幸福美好金张掖的决战决胜期，也是全面深化改革、创新驱动发展的攻坚克难期。深入分析张掖发展的阶段性特征和整体性特点、外部环境和内部条件，机遇千载难逢、殊为难得，挑战复杂多变、十分严峻。我们必须牢牢把握经济社会发展的趋势性变化，因势利导、顺势而为，趋利避害、化危为机，主动作为、乘势而上，再鼓实干之劲、再造发展之势、再聚和谐之力，努力开创经济社会发展新局面。

政府工作的总体要求是：全面贯彻党的十八大和十八届三中、四中、五中、六中全会精神，深入贯彻习近平总书记系列重要讲话精神和治国理政新理念新思想新战略，按照中央“四个全面”战略布局和省委、省政府“八个并重”“六大支撑”等部署要求，认真贯彻落实市第四次党代会精神，坚持“坚定贯彻‘五大发展理念’，围绕‘四二’部署，实施‘六六’战略，加快建设幸福美好金张掖”的基本思路，以全面深化改革和创新驱动发展为根本动力，以实现更高发展质量和更好经济效益为中心，以推进供给侧结构性改革为主线，加快转方式调结构促升级惠民生步伐，统筹推进经济建设、政治建设、文化建设、社会建设、生态文明建设，为率先在全省全面建成小康社会而努力奋斗。

经济社会发展的主要目标是：生产总值年均增长8%以上，突破600亿元。固定资产投资年均增长15%，突破700亿元。大口径财政收入和一般公共预算收入年均增长10%，达到100亿元和45亿元。招商引资到位资金年均增长15%，累计达到3000亿元以上。城乡居民人均可支配收入年均增长8%和9%，达到31200元和18200元。贫困发生率控制在1%以内，全面小康实现程度达到90%以上。三次产业结构调整到18∶32∶50，非公经济比重提高到65%以上。综合经济实力、城乡建设水准、公共服务能力、人民生活质量显著提升。

主要任务是：

（一）培育富有特色和市场竞争力的产业体系，建设丝绸之路西部创新型城市。加快河西走廊科技中试和成果转化基地、张掖科技大市场、创业孵化综合科技服务平台建设，推动创新资源有效集聚、创新活力充分激发、创新主体大量涌现、创新能力不断增强，力争市场主体总量达到15万户。建成全国小微企业创业创新基地示范城市、省级科技创新型试点城市和可持续发展实验区，高新技术企业达到50家，万人口发明专利拥有量达到2.5件，科技对经济增长的贡献率提高到55%以上。坚持循环型生态工业发展方向，持续完善“2+4”工业园区支撑体系和“一特两新”产业体系，重点发展绿色有机食品加工业，做强风光电、生物质能发电等新能源产业，做优矿产化工新材料产业，培育壮大生物医药、装备制造、通用航空、新型煤化工、节能环保等战略性新兴产业，建成河西走廊经济区集聚辐射带动能力较强的中心城市。大力实施互联网+“中国制造战略，推动新型工业化、信息化、城镇化和农业现代化互动发展。突出科技创新，强化管理创新、制度创新和商业模式创新，用创新驱动改造提升传统产业，构建以创新为主要驱动力的经济体系和发展模式。

（二）打造绿色有机农产品生产基地，建设国家现代农业示范市。以“三元双向”循环为基本模式，深入实施“四个百万”工程，大力推进国家农业科技园区、国家玉米制种基地、全国农业农村信息化示范基地、全国粮改饲和草牧业试点、全省现代畜牧业全产业链试点市建设，努力提升农业整体效益和市场竞争力。适应市场需求变化，积极发展观光型、体验型等功能型农业。健全农业投入稳定增长机制，确保年增速保持在20%以上。大力引进推广高新技术和科技成果，强化农业规模化、标准化、品牌化生产，加快发展龙头骨干企业，着力培育新型经营主体，提升主导产业规模效益和辐射带动能力，建成国家绿色有机农产品生产基地和生态养殖业示范基地。

（三）实施旅游文化体育医养融合发展战略，建设中国全域旅游典范城市。按照全域、全要素、全产业链发展的目标，抓住城乡规划、交通建设、环境整治等关键环节，持续开展“四大行动”，推动旅游景观全域优化、旅游服务全域配套、旅游产业全域联动。实施旅游与文化、体育、医养融合发展战略，加快构建形成完整的产业链和复合型产业集群。着力打造丝绸之路黄金旅游线重要目的地、中国西部区域游客集散中心，建成国家全域旅游示范区。接待游客突破3300万人次，综合收入突破220亿元，直接就业8万人以上，把旅游业培育成富民强市的战略性支柱产业。加快发展以电子商务、现代物流、金融服务、健康养老为重点的新兴业态，挖掘消费潜力，强化消费拉动，着力提高现代服务业发展的质量效益，培育形成新的消费热点和经济增长点。

（四）构建要素完善支撑有力的开发格局，建设河西走廊经济区枢纽城市。深入实施“6873”交通突破行动，加快“四横九纵十三联”路网建设，建成兰州至张掖客运专线和张扁高速、张肃一级等重大交通项目，实现县县通高速、乡镇通国省道、村村通硬化路、级旅游景区和重点产业园区通二级以上公路。完成张掖机场二期和5个左右通用航空机场（起降点）建设任务，形成东西畅通、南北贯通、互联互通、立体交通格局。完善提升滨河新区承载服务功能，争取试点建设地下综合管廊和海绵城市。加快市政设施和智能交通系统建设，推进城市特色风貌和棚户区、城中村改造，市区和五县城、12个重点镇实现集中供热供水供气、污水垃圾处理全覆盖，城镇化率达到50%以上。加快实施“6363”水利保障行动，完成黑河张掖城区段治理任务，实施灌区节水配套改造、河道治理、水资源综合利用、农村饮水安全巩固提升等重点项目，增强基础支撑保障能力。加快电力“1569”工程建设，建成750千伏变电站，新改建330、110千伏变电站15座，提高供电保障和电力外送能力。推进骨干城域网扩容等信息基础工程，提升网络容量和传输能力，出口带宽提高到以上。加快云计算发展，建设区域性大数据中心，打造“数字张掖”，基本建成“智慧城市”。

（五）营造包容开放公平有序的发展环境，建设向西开放门户城市。充分发挥张掖区位、交通、资源、产业方面的优势，面向中西亚、东欧等国际市场，积极建设向西开放的桥头堡和大通道，推动农产品和农业技术、农机装备、专业人才开拓国际市场，加快农业“向西走出去”步伐。深入实施“引强入张”战略，持续扩大招商引资、引智引创，实现招商引资到位资金、引入500强企业、引进项目“三个大幅增长”，以招商形成的产业、产能、产品支撑“向西走出去”。配套完善张掖国家级经济技术开发区、民乐生态工业园区、张掖国际物流园区、绿洲现代物流园区基础设施，集聚周边优质特色产品积极开拓“一带一路”大市场。自营进出口企业、出口产品、进出口总额分别达到50家、50万吨、5亿美元以上。加强与周边省区和河西五市间的合作交流，着力构建“2小时”经济圈。提升“张交会”市场化、专业化程度和国际化水平，打造丝绸之路经济带最具影响力的品牌节会。全面改善营商环境，提升政务服务、社会服务能力水平，为大招商、大开放、大发展创造优良条件。

（六）巩固提升生态环境的比较优势，建设西部生态安全屏障市。全面落实国家主体功能区和甘肃生态安全屏障综合试验区规划，加大祁连山、黑河湿地国家级自然保护区保护治理力度，争取实施祁连山生态保护与建设、黑河流域综合治理规划。建立重点生态功能区产业准入负面清单，合理控制开发强度。强化黑河国际重要湿地履约管理工作。实施新一轮退耕还林还草工程，落实草原生态补奖政策，加快建设生态补偿试验示范区和张掖走廊生态带、黑河大林带。开展“百万亩造林绿化行动”，森林覆盖率达到17.5％以上。实行最严格的环境保护制度，持续开展大气、水、土壤污染防治和城乡环境综合整治，深入推进节能降耗，倡导绿色消费、低碳消费，促进资源节约循环利用，单位生产总值能耗、主要污染物排放等约束性指标控制在限额以内。加快国家生态文明示范区建设，构筑安全可靠的生态屏障，巩固扩大优良生态、优美环境在支撑发展方面的比较优势。

（七）着力提高人民生活水平，率先实现全面小康目标。聚焦全面建成小康社会，着力补齐短板，力争主要指标争先进位，确保农村居民人均可支配收入继续保持全省领先水平，确保城镇居民人均可支配收入增幅高于全省平均水平，确保率先在全省建成全面小康。着力培植骨干财源，实现财政收入“三个高于”增长目标。全面完成易地扶贫搬迁任务，实现水、电、路、讯、房等基础设施全覆盖，力争2020年扶贫对象人均可支配收入接近全市农村居民平均水平。积极推进学前教育扩容增量、义务教育均衡达标、高中教育普及提质、职业教育加快发展，支持河西学院创建河西大学，促进各类教育协调发展。着力构建公共文化服务和现代文化市场体系，促进公共文化服务标准化、均等化。实施全民健身国家战略，推动体育产业和体育事业协调发展。完善就业、养老、医疗、工伤、失业和生育等保障制度，统筹推进社会救助体系建设。加快发展医疗卫生事业，“健康张掖”建设取得明显成效。丰富甘州全国综合养老示范基地建设内涵，带动发展养老产业。持续加强法治张掖、平安张掖、和谐张掖建设，积极推进公安改革，全面提升社会治理水平和人民群众的安全感、幸福感。

2017年工作重点

2017年，是新一届政府履职的开局之年，做好各项工作至关重要。主要预期目标是：生产总值增长8%。固定资产投资增长13%。社会消费品零售总额增长10%。大口径财政收入和一般公共预算收入均增长10%。城乡居民人均可支配收入分别增长8%和9%。单位生产总值能耗、主要污染物排放等约束性指标控制在省上下达指标内。

重点抓好七个方面的工作：

（一）强化项目带动，激活发展要素，促进经济持续稳定增长

突出项目引擎地位，坚持实行重点项目“五个一”推进机制，严格落实工作责任，用大项目、好项目促投资、保增长、惠民生。力争全年开工建设各类项目600项以上，完成固定资产投资399亿元。

积极谋划争取实施一批重大项目。主动对接国家产业政策、投资取向，深入谋划论证和争取实施生态保护、基础设施、公共服务及主导产业发展等方面的项目，努力使更多项目进入国家和省上计划盘子。紧抓国家支持发展战略性新兴产业、省上整合资金10亿元每年支持100个重点项目的政策机遇，在新能源及装备制造，钨钼等冶金新材料，中药材、牛羊肉、食用菌、红枣、沙棘、枸杞等特色农产品深加工，先进农业机械制造、新型煤化工等方面争取更多项目支持。加快推进盘道山抽水蓄能电站等重大项目前期工作，力争早日落地建设。

持续扩大招商引资和民间投资。拓展招商引资领域，创新招商引资方式，强化以商招商、精准招商、节会招商，确保招商引资项目数量、签约资金、完成投资持续增长，到位资金增长20%，突破600亿元。进一步完善招商项目落地运行机制，强化全程跟踪服务，加快手续办理进度，协调解决困难问题，促进招商项目早开工、早建设。大力推广PPP模式，选准项目科学谋划，有序推进特许经营，建立多维度、全覆盖的市县两级项目库，做好重点示范项目申报争取工作。设立PPP投资引导基金，激活社会资本、民间投资，争取开工建设一批示范项目。

不断拓宽投融资渠道。充分发挥信贷在资源配置中的重要作用，支持金融机构创新服务方式，鼓励商业银行开展投贷联动试点，积极引进银行、保险、证券、基金等优质金融资源驻张设点，进一步提升实体经济发展的支撑力。紧抓农发行、国开行政策敞口有利时机，在易地扶贫搬迁、水利交通、棚户区改造等项目上争取更多更大信贷支持。探索支持小微企业创新创业融资新途径，重点发展多层次资本市场，鼓励企业发行标准化债权产品，推动符合条件的企业在新三板挂牌融资。全面落实绩效考核管理办法，提升政府投融资平台资本运作水平，力争完成投资60亿元以上。积极推进张掖经济技术开发区15亿元企业债券发行工作，启动市山水文旅投股改试点。加强甘肃旅游产业投资基金管理运营，进一步放大投资效应。

大力培育新的经济增长点。深度融入丝绸之路经济带建设，加强与周边地区合作，促进优势互补、联动发展，不断提升“向西开放”水平。深化与霍尔果斯中哈边境合作中心和国家经济技术开发区的战略合作，支持企业出口绿色有机农产品，争取在中西亚地区建立农产品生产加工销售基地。做大做强电子商务产业园，培育壮大农村电商市场主体，支持企业和金融机构发展电商交易、第三方支付、物流配送、电商代运营等衍生服务业。加快张掖国际物流园、绿洲现代物流园等重点物流园区建设，积极筹建快递物流园，着力打造西部物流集散基地和商贸流通中心。

（二）打造产业集群，延长产业链条，提升主导产业质量效益

聚焦重大产业项目，推动主导产业提质增效，促进三次产业资源共享、融合发展、转型升级，实现有质量、高效益、可持续发展。

以集约化、产业化、标准化为导向，以“三元双向”循环为基本模式，加快发展现代农业。提升国家玉米制种基地建设管理水平，建设种业市场监管“首善之区”，加快种子企业联合上市步伐。持续推进现代畜牧业全产业链建设，新建标准化规模养殖场（区）100个，争创省部级标准化示范场5个，肉牛、奶牛、肉羊饲养量分别达到105万头、6万头和580万只。加快发展食用菌产业，新增产量1万吨。力争建成三元乳业、金阳光食品等14个重点农业产业化项目，完成投资13亿元以上，新增农产品加工能力20万吨以上。大力发展新型农业经营主体和标准化生产，新培育示范性专业合作社150家，新认证“三品一标”农产品10个以上，建立标准化示范点20个。实施农民素质提升工程，完成农村劳动力转移就业技能培训3.3万人。输转劳动力28万人以上，实现劳务收入50亿元以上。

以培植财源为重点，以园区建设为载体，提升工业经济发展水平。加快建立覆盖三次产业，特别是以第二产业为主要基础、二三产业为基本支撑的财源体系。围绕构建“一特两新”产业体系，加快“2+4”工业园区平台建设，完善道路、供水、供气、排污等配套设施，提升基础设施、公共服务覆盖面和共享度。开工建设重点工业项目120项，其中亿元以上34项，完成投资74亿元以上。着力壮大以畜产品、食用菌、果蔬、乳制品为重点的绿色有机食品加工产业集群，延长精深加工循环产业链。全力推进花草滩2×35万千瓦低热值煤发电、兰州能投20万吨中温焦油加氢、滨河高原生态科技文化创意产业园等项目建设，确保高崖子滩二期330千伏升压站等重点项目建成运行。强化要素保障，加大协调服务，确保建成项目80%以上达产达标，争取巨龙、华兴等7家铁合金企业纳入全省直购电交易平台。新增规上企业16户，工业增加值增长8.5%以上。

以全域旅游为引领，以深化“四大行动”为抓手，促进旅游业提档升级。坚持“文化为魂、旅游为体、体育为用、医养为本”的发展理念，深度整合、综合利用特色资源，加快建设国家全域旅游示范区和全省旅游文化体育医养融合发展示范区。持续深化“四大行动”，积极申报祁连山国家公园和丹霞世界地质公园、世界自然遗产，着力提升丹霞大景区建设水平，力争进入国家级景区创建名录。完成弱水花海、丝绸之路文化遗产博览城等36项续建项目年度任务，开工建设丹霞轨道交通、峡谷奇观等6个重点旅游项目，完成投资43亿元以上。加快推进与港中旅、长城影视等知名旅游文化企业合作进程。完善市场化运作机制，持续扩大中国·张掖汽车拉力锦标赛、祁连山国际超百公里山地户外运动挑战赛等品牌赛事的对外影响力。办好第二届通航大会。积极开发登山探险、滑翔飞行、低空观光等特色旅游产品，加快建设国际户外运动名城，不断提升旅游业核心竞争力。建成张掖旅游大数据中心及智慧旅游服务、管理和营销平台。加强旅游标准化体系建设，健全完善多部门联合执法检查、旅游投诉和突发事件应急处理、旅游行业自律等机制，推动旅游市场治理规范化常态化长效化。力争全年接待游客增长20%以上，达到2500万人次，旅游综合收入达到125亿元，旅游对经济增长的支撑和带动能力持续增强。

（三）加快落实“双创”政策，激发市场主体活力，开创大众创业万众创新新局面

坚持就业优先基本方向，突出改革创新根本动力，以最优惠的政策、最优质的服务、最优良的环境推进创新创业，全面完成“双创”示范建设任务，增强发展新动能，增创发展新优势。

全面落实地方政策，激发全民创新创业热情。立足降低创业风险和成本，全面落实“黄金19条”优惠政策，健全完善张掖版的融资服务体系，破解融资瓶颈，推动创新创业。修订完善初创型小微企业“五免一补”、成长型小微企业“五奖两补”、创业创新基地“两补四奖”等扶持政策，最大程度发挥政策性资金的撬动作用。通过减免新入驻企业租金、补贴示范基地运营费和发放科技专项奖励、创新奖励及专利扶持资金等措施，促进小微企业投资持续大幅增长。加大“双创贷”“助创贷”“助保贷”等金融产品和天使投资、科技创新创业投资等基金投放力度，形成创新资源和创新活力纷涌流入的“洼地”效应，激发释放“双创”活力。

基本建成“双创”示范基地，健全完善服务保障体系。坚持“布局合理、错位互补、集约高效、特色鲜明”的总体思路，加快35个众创空间和示范基地建设，建成各类创业创新基地100万平方米。丰富提升民乐中药材产业创新创业孵化园、张掖创意文化产业园、现代农业孵化园等14个创业平台建设内涵，新认定省级以上众创空间和孵化器7家以上、市级8家以上。提高西部创客大学办学水平，强化“双创”服务保障。大力引进咨询、设计、财务、证券等第三方服务机构，新建16家小微企业公共服务平台，力争新入驻基地的小微企业和创新团队达到1000家以上。

着力实施“三个一批”工程，提升科技支撑能力。充分发挥企业科技创新主体作用，增强中小微企业创新能力和创造活力，促进新技术、新产业、新业态加快发展。加大税收、租金、贷款、担保等政策倾斜力度，培育扶持一批科技型企业，力争新发展高新技术企业10家、省级技术中心5家。创新服务模式，强化政策支持，加快转化一批科技成果，力争新增专利授权500件以上，新转化各类科技成果50项以上，技术合同交易额达到18亿元以上。全面落实人才政策，深化专业技术人员职称制度改革，积极培育引进一批高科技人才，有效发挥专家工作站和科技特派员作用，进一步加强科技创新人才和企业经营管理人才队伍建设，推动技术创新和发展方式转变。

（四）加快完善城乡基础设施，强化规划建设管理，全力推进新型城镇化进程

突出产业聚集，全力补齐短板，完善承载功能，彰显地域特色，形成宜居宜游、宜商宜业，城乡互补、协调发展的新局面。

配套完善城市基础设施。加快市区及五县城区道路交通、市政基础设施、园林绿化等公共服务设施建设，持续提升综合服务功能。实施市区道路畅通工程，打通新老城区所有“断头路”，启动西南二环路综合改造工程。建设市区城市生活饮用水水质检测中心、集中供热老旧管网改造和市污水处理厂一期提标技改等重点市政工程，确保垃圾焚烧发电项目建成运行。继续推进市区既有建筑节能改造和燃煤锅炉拆并工作，集中供热扩容200万平方米。扎实推进城市慢行系统、立体停车场、城市充电桩和便民市场建设。深化住房制度改革，坚持购租并举，加快培育和发展住房租赁市场。引导房地产企业发展精品特色地产，制定优惠政策吸引周边地区居民来张购房置业、居住养老，促进房地产市场平稳健康发展。准确定位和塑造城市建筑文化，以“清雅甘州”为主题持续推进风貌改造，全面展示丝绸之路历史记忆。加快滨河新区建设，进一步完善供水供热供气、排污防涝、电力通信等各类地下管网及路网框架，持续配套教育、文化、医疗、养老等公共服务设施，实现重大市政基础设施和公共服务全覆盖。加大产业孵化培育力度，引导产业向新区集中、集约发展，促进人流、物流、信息流、资金流向新区集聚，促进新老城区融合发展。

提升城市规划建设管理水平。突出城市内涵式发展，按旅游城市标准，统筹本地居民需求与游客便利，促进各类优质资源共建共治共享。尊重城市发展规律，巩固推广高台“多规合一”试点工作成果经验，构建科学完备的城乡规划体系。综合运用土地资本、地域空间及其它经济要素，盘活土地资产存量，建立多元、可持续的城市建设投融资机制。加大棚户区改造、老旧住宅区改建和棚户区货币化安置力度，确保能改则改、应纳尽纳，努力改善群众居住环境和生活条件。加快民乐、山丹创建省级园林县城步伐，巩固提升临泽、高台国家园林县城创建水平，确保市区成功创建国家园林城市。加强城市综合执法，规范完善社区服务和物业管理，提高城市精细化管理水平。开展城乡环境、违章建筑、旅游市场、交通秩序四项重点整治，持续改善城市形象。深入开展公民道德素质教育和精神文明创建活动，不断提升全社会文明程度。

加快建设美丽乡村。扎实推进12个国家级重点镇、35个“特色小镇”、12个省级“千村美丽”示范村建设，加快路网、管网、电网、通讯网集成配套。积极探索符合农村实际的污水排放和垃圾处理方式，推广新型节能环保采暖设施，建立健全生态环境、村容村貌、公共卫生管理长效机制，培养文明健康生活方式，不断改善农村生产生活条件。统筹文化内涵、建筑艺术、产业支撑等元素，挖掘培育美丽乡村特色优势，做到县县有品牌、乡乡有样板、村村有特色，建设环境优美、生态宜居、特色鲜明、幸福和谐新农村。

（五）加强生态环境保护，改善发展条件，持续增强发展的动力和后劲

健全完善保护生态、改善生态、永续发展的体制机制，扩大生态优势，建设宜居家园。

加快祁连山和黑河湿地自然保护区建设。认真组织实施山水林田湖生态修复、黑河湿地湖泊治理与保护、黑河城区段治理、天然林保护、三北五期防护林、防沙治沙、新一轮退耕还林还草、草原生态补奖等重点工程，争取实施张掖走廊生态带和黑河大林带建设规划、丝绸之路经济带黑河流域（张掖段）湿地生态走廊建设规划及自然保护区基础设施建设、黑河湿地保护与恢复、国际重要湿地生态效益补偿试点等重点生态项目，提升祁连山和黑河湿地国家级自然保护区建设质量和水平。继续开展大规模植树造林，新增造林20万亩。

持续改善环境质量。紧抓产业结构布局、能耗减排、道路扬尘和机动车尾气排放等关键环节，建立大气环境污染物监测预警网络，推动大气污染防治向多因子、全方位、区域协同控制转变，力保优良天数达标。加强水生态保护和水资源管理，优先保护饮用水水源地，强化工业生产等重点领域水污染防治，确保水环境安全。加大城镇水污染防治力度，整治市区饮马河、东环路芦苇池、东泉干渠、黄水沟4条黑臭水体。推进重点领域、重点行业污染减排，积极开展排污权有偿使用和交易试点，确保工业污染源达标排放。大力开展农村面源污染治理，提升废旧农膜、种养业副产物等农业废弃物资源化综合利用水平，减少农村环境污染。坚持“防、控、治”并重，强化源头监督管理，积极推进土壤污染治理修复，逐步改善土壤质量。

扎实推进交通水利电力信息化建设。加快张扁高速、甘临一体化一级公路、G312线马营口至山丹西、G213线肃南至祁连、六东一级公路二期、S590线山丹至马场二场公路、高台至大湖湾景区等国省道和旅游公路建设进度，力争张肃一级、金昌至山丹二级等公路项目立项建设。建成张掖综合枢纽站、丹霞景区客运站，力争开工建设山丹新城区客运站和肃南客运站。持续加强农田水利建设，完成投资15亿元。继续实施河西走廊国家级高效节水灌溉项目，新发展高效节水灌溉面积15万亩。改建供水工程12处，解决1万人饮水安全不稳定问题。力争开工建设酥油口、山水河等小型水库。整理土地6万亩，新建高标准农田10万亩。开工建设750千伏张掖输变电及330千伏甘州变项目。持续实施光缆扩容传输等重点通讯工程，加快推进沿祁连山区、“插花型”扶贫片区及民族地区信息化建设，提升“宽带中国”示范城市建设水平。

（六）加大民生保障投入，改善公共服务条件，提高群众幸福感和满意度

坚持建机制、补短板、兜底线，积极回应群众关切，繁荣社会事业，创新治理方式，维护公平正义，让人民群众有更多获得感。

完善社会保障体系。坚持以创业带动就业，城镇新增就业2.4万人以上，城镇登记失业率控制在2.6%以内，高校毕业生就业率达到85%以上。积极推进社会保障制度改革，加快城乡居民基本医疗保险制度整合，提高社会保险参保率，进一步扩大基金规模。加大社会救助力度，保障困难群众基本生活。加快建设甘州全国综合养老示范基地，建成市民政精神病福利院，新建城乡社区老年日间照料中心80个。改造棚户区17954套（户）。高度重视残疾人兜底保障工作，建成市级和临泽、山丹残疾人托养中心及市级和民乐残疾人康复中心。全面落实“两规划”指标，维护妇女儿童合法权益。

加大教育医疗投入。认真实施“全面改薄”、行政村幼儿园、农村教师周转宿舍等重点项目，确保张掖二中分校、民乐思源实验学校建成招生，完成培黎国际职业学院年度建设任务，开工建设甘州思源实验学校和市电大、党校新校区。巩固提升医疗卫生机构标准化创建成果，基本建成市第二人民医院。积极推进公立医院综合改革，加强重大疾病防控，提高医疗质量和基本公共卫生均等化服务水平。落实计划生育优惠政策，促进人口均衡发展。

提高公共文化服务水平。深化华夏文明传承创新区建设，巩固提升国家公共文化服务体系示范区创建成果，建设市级数字“三馆”和非物质文化遗产保护传承中心，积极筹建市科技馆，确保张掖大剧院和民乐“五馆”、山丹“七馆”、临泽“三馆三中心”建成运营。推进“历史再现”工程，新建博物馆5座、“乡村记忆”博物馆10座。加强历史文化遗存传承保护，完成西来寺、土塔和黑水国遗址等8项文物修缮保护工程。加强精品文艺创作。启动实施国际户外运动名城建设规划，加快市级大型体育场馆、张掖国家登山健身步道、城市社区15分钟健身圈、全民健身路径等体育惠民工程建设。

扩大精准扶贫脱贫成果。坚持精准扶贫脱贫与区域发展统筹谋划、与优势产业培育紧密结合、与社会保障有效衔接，推动精准扶贫向小康建设转变。实施好易地扶贫搬迁项目，全面完成1142户3922人安置任务，开工建设3106户10029人易地扶贫搬迁工程。继续整合资源，加大资金投入，突出发力重点，确保精准扶贫取得决定性胜利。

维护社会大局稳定。制定安全生产责任清单和权力清单，建立完善安全生产“五级五覆盖”责任体系，抓好重点行业专项整治，有效防范和坚决遏制重特大生产安全事故发生。严格食品药品监管，全面落实“四个最严”和“四有两责”要求，确保人民群众饮食用药安全。加强价格监管，保持物价基本稳定。加强全民国防教育，推进军民深度融合发展。强化网格化管理，创建和谐社区。进一步完善应急管理体系，提高防灾减灾救灾能力。坚持问题导向，打造“阳光信访”，全力化解社会矛盾。健全社会治安防控体系，深入开展平安创建活动，严密防范和依法打击各类违法犯罪活动，维护社会和谐稳定。

（七）加快职能转变，严格依法行政，全面加强政府自身建设

新的形势、新的任务、新的征程，对政府工作提出了新的更高要求。我们将以更加强烈的责任担当和更加务实的工作作风，运用法治思维和法治方式深化改革，持之以恒加强政府自身建设，切实推进治理体系和治理能力现代化。

提高“放管服”水平。按照“能放全放、能减全减，该管严管、善管会管”的要求，推动简政放权、放管结合、优化服务改革向纵深发展。完善“三张清单一张网”运行机制，强化行政审批事中事后监管，实现“宽进严管”，不断提升行政效能和服务水平。深入实施权责清单制度，清理公开公共服务事项目录，加快推进“网上行权”，进一步规范部门单位行政行为。深化环评审批制度改革试点，认真总结经验，推进流程再造，提升服务效能。大力推行“互联网+政务服务”，促进部门间公共数据共享，让群众少跑腿、好办事。推进政府新闻发布制度化，提高政务公开水平。

深化重点领域改革。按照“降低制度性交易成本、降低政府行政运行成本、降低公众创业创新成本，提升全社会创造活力”的原则，完善制度设计，创新方式方法，确保重点领域和关键环节改革取得新突破。深入推进商事制度改革，全面落实全程电子化登记管理及“一照多址”“一址多照”、集群注册等改革措施，加快实施“五证合一”“一照”“双告知”“双随机一公开”改革，积极推行企业、个体工商户简易注销程序改革，实施个体工商户“两证”整合。全面落实减税降费让利等措施，降低企业生产经营成本，大力推进“个转企、小升规、规改股、股上市”，力争“个转企”达到1000户以上。完成市县两级国有企业改革任务。创新土地、用能、排污权和企业产权、股权、租赁权等要素配置方式，实现资源效益最大化。积极开展农村产权交易和产权抵押贷款，推动农村土地承包经营权、农业设施产权和农民住房财产权等质押贷款取得明显成效。推进农村土地所有权、承包权、经营权“三权”分置，确保农业健康发展和农村社会稳定。完善政府购买服务制度和服务平台机制，扩大政府购买公共服务范围。统筹加快公共卫生、农林水牧、交通运输、文化旅游、资源环境、安全生产、城乡建设管理等领域执法改革，创新执法机制，推行综合执法，提升执法水平。

严格依法行政。大力推进民主法治建设，严格按照法定权限和程序履行职责，切实做到法定职责必须为、法无授权不可为。严格实行重大决策公众参与和专家咨询制度，加强社会稳定和行政决策风险评估，坚持政府法律顾问制度和全程跟踪反馈、终身责任追究制度，规范各项工作程序，促进政府工作制度化、规范化。扎实开展“七五”普法，努力推动形成办事依法、遇事找法、解决问题用法、化解矛盾靠法的良好环境。深入开展民族团结进步创建活动，提高宗教工作法治化水平。主动接受人大法律监督和工作监督、政协民主监督、新闻舆论监督、社会各界和人民群众监督，保证人民赋予的权力始终用来为人民谋利益。

加强纪律作风建设。深入贯彻落实全面从严管党治党的总体部署和市委“八项工作要求”，进一步加强政府党组建设。切实承担起党风廉政建设主体责任，坚决查处以权谋私、公权私用以及违反中央八项规定精神的违纪违规行为，建设廉洁型政府。规范“三公”经费等一般性支出，强化公务卡结算管理，把艰苦奋斗、厉行节约体现在政府运行各环节、各方面。坚持把纪律挺在前面，用铁的纪律规范和加强政府系统党组工作，进一步强化责任意识、服务意识、效能意识，推动作风建设常态化。持续治理行政不作为、乱作为等问题，对制定的政策、确定的目标、部署的任务一抓到底，做到事有专管之人、人有明确之责、责有限定之期，以严明纪律畅通政令，以工作实绩展示执行力、提升公信力。

各位代表，全面建成小康社会的宏伟蓝图，承载着130万张掖人民的梦想和荣光。满足人民群众过上更好生活的新期待，我们深感使命神圣、重任在肩，唯有更加勤勉尽责、奋发努力，才能不负重托、不负众望。让我们紧密团结在以习近平同志为核心的党中央周围，坚决贯彻落实省委、省政府决策部署，在市委的坚强领导下，以更大的勇气改革创新，以更强的毅力攻坚克难，以更实的作风干事创业，为加快建设幸福美好金张掖、率先在全省全面建成小康社会而努力奋斗！
